# Complete Genome Sequence of a Bovine Coronavirus Isolated from a Goat in Pennsylvania, USA

**DOI:** 10.1128/mra.00122-23

**Published:** 2023-05-03

**Authors:** Shubhada K. Chothe, Maurice Byukusenge, Manoj K. Sekhwal, Lingling Li, Lindsey Cecelia LaBella, Padmaja Jakka, Kay Palchak, Rhiannon Barry, Michele Yon, Ruth H. Nissly, Kathleen M. Kelly, Bhushan M. Jayarao, Meera Surendran Nair, Suresh V. Kuchipudi

**Affiliations:** a Pennsylvania State Animal Diagnostic Laboratory, Department of Veterinary and Biomedical Sciences, Pennsylvania State University, University Park, Pennsylvania, USA; b Huck Institutes of the Life Sciences, Pennsylvania State University, University Park, Pennsylvania, USA; DOE Joint Genome Institute

## Abstract

We report a complete genome sequence of bovine coronavirus (BCoV) isolated from a goat in the state of Pennsylvania in 2022. BCoV often causes calf scours and winter dysentery in cattle.

## ANNOUNCEMENT

Bovine coronavirus (BCoV) is an important livestock pathogen that causes respiratory and enteric infections in cattle and wild ruminants (1). BCoV belongs to the species *Betacoronavirus 1* and the family *Coronaviridae*, which includes viruses such as equine coronavirus, porcine hemagglutinating encephalomyelitis virus, and severe acute respiratory syndrome (SARS)-related coronaviruses. BCoV is an enveloped virus possessing a single-stranded, positive-sense, nonsegmented RNA genome of around 31 kb. While BCoV is associated with neonatal calf diarrhea, winter dysentery, and respiratory disease in adult cattle (2), little is known about the effects of BCoV in domestic goats in the United States. A previous study reported BCoV PCR-positive fecal samples in an adult dairy goat during a diarrhea outbreak (3). However, no complete BCoV genome sequence isolated from a small ruminant is currently available in the public database.

In this study, we report a complete and assembled genome sequence of BCoV isolated from the lung tissue of a 2-year-old goat (Capra hircus) in Pennsylvania, USA. The lung sample was collected in April 2022 and submitted to the Penn State Animal Diagnostic Laboratory (ADL) from a veterinary practice in Pennsylvania. Gross and histopathological investigations at ADL revealed acute interstitial pneumonia and bronchiolar necrosis, consistent with coronaviral pneumonia. RNA was extracted from the homogenized lung tissue using the MagMAX pathogen RNA/DNA kit (Thermo Fisher Scientific, MA) and subjected to BCoV-specific reverse transcription-PCR (RT-PCR), using the TaqPath 1-step RT-quantitative PCR (qPCR) master mix (Thermo Fisher Scientific) and primer and probe set previously described (4). Upon positive PCR identification, the lung tissue homogenate was further inoculated into a human colon cancer cell line (HRT-18G; ATCC, VA) supplemented with TPCK (tosylsulfonyl phenylalanyl chloromethyl ketone)-treated trypsin (2 μg/mL; Thermo Fisher Scientific) for virus isolation. The cells showed cytopathic effects at 5 days postinoculation. The cell culture supernatant was utilized for viral RNA extraction using the Quick-RNA viral kit (Zymo Research, USA). The TruSeq stranded total RNA and the Ribo-Zero Plus rRNA depletion kit (Illumina, USA) were used to prepare a sequencing library, and sequencing was performed on the Illumina MiniSeq platform, generating 6,056,928 paired-end reads of 150 bp each. The raw sequence reads were trimmed for quality and to remove adapters using the FASTP program (5). A *de novo* genome assembly was performed using the Unicycler v.0.4.8 program (6). Unicycler was used as a SPAdes optimizer with only short reads as the input. The assembled contigs were subjected to a BLAST search against the nucleotide database using the BLASTN v.2.9.0 program (7). Only one contig of 31,078 bp had a hit corresponding to a viral genome. The assembled genome had an average coverage depth of 7,425× and a GC content of 36.98%. It showed a 99% nucleotide identity to a cattle-associated bovine coronavirus genome (GenBank accession number MH043954) that was previously isolated in Pennsylvania (8). BWA-MEM v.0.7.17 (9) was used to map the reads to this genome as the closest relative, and Freebayes v.0.9.21 (10) was used to identify the variants. A 10-nucleotide deletion within the 4.9-kDa nonstructural protein was identified. There were also 290 single nucleotide variants (SNVs), of which 53.8% and 34.5% were within the spike- and ORF1ab-encoding genes, respectively. The precise biological implications of the observed variations in the spike- and ORF1ab-encoding genes are unknown and warrant further functional studies.

The Viral Annotation Pipeline and Identification (VAPiD) v.1.6.7 tool (11) was used to annotate the assembled genome using the NCBI reference sequence (GenBank accession number NC_003045.1). All tools were run with default parameters unless otherwise specified.

### Data availability.

The complete genome sequence was deposited at NCBI GenBank under accession number OP004056. The associated short reads were submitted to the Sequence Read Archive (SRA) under the accession number PRJNA946767.

## References

[1] Saif LJ. 2010. Bovine respiratory coronavirus. Vet Clin North Am Food Anim Pract 26:349–364. doi:10.1016/j.cvfa.2010.04.005. 20619189 PMC4094360

[2] Amer HM, Abd El Wahed A, Shalaby MA, Almajhdi FN, Hufert FT, Weidmann M. 2013. A new approach for diagnosis of bovine coronavirus using a reverse transcription recombinase polymerase amplification assay. J Virol Methods 193:337–340. doi:10.1016/j.jviromet.2013.06.027. 23811231 PMC7113639

[3] Smith FL, Heller MC, Crossley BM, Clothier KA, Anderson ML, Barnum SS, Pusterla N, Rowe JD. 2022. Diarrhea outbreak associated with coronavirus infection in adult dairy goats. J Vet Intern Med 36:805–811. doi:10.1111/jvim.16354. 35165938 PMC8965271

[4] Cho Y-I, Kim W-I, Liu S, Kinyon JM, Yoon KJ. 2010. Development of a panel of multiplex real-time polymerase chain reaction assays for simultaneous detection of major agents causing calf diarrhea in feces. J Vet Diagn Invest 22:509–517. doi:10.1177/104063871002200403. 20622219

[5] Chen S, Zhou Y, Chen Y, Gu J. 2018. fastp: an ultra-fast all-in-one FASTQ preprocessor. Bioinformatics 34:i884–i890. doi:10.1093/bioinformatics/bty560. 30423086 PMC6129281

[6] Wick RR, Judd LM, Gorrie CL, Holt KE. 2017. Unicycler: resolving bacterial genome assemblies from short and long sequencing reads. PLoS Comput Biol 13:e1005595. doi:10.1371/journal.pcbi.1005595. 28594827 PMC5481147

[7] Camacho C, Coulouris G, Avagyan V, Ma N, Papadopoulos J, Bealer K, Madden TL. 2009. BLAST+: architecture and applications. BMC Bioinformatics 10:421. doi:10.1186/1471-2105-10-421. 20003500 PMC2803857

[8] Byukusenge M, Nissly RH, Kasibhatla SM, Li L, Russell R, Springer H, Barry R, Van Saun R, Wolfgang D, Hovingh E, Kulkarni-Kale U, Kuchipudi SV. 2018. Complete genome sequences of four bovine coronavirus isolates from Pennsylvania. Genome Announc 6:e00467-18. doi:10.1128/genomeA.00467-18. 29853507 PMC5981048

[9] Li H, Durbin R. 2009. Fast and accurate short read alignment with Burrows-Wheeler transform. Bioinformatics 25:1754–1760. doi:10.1093/bioinformatics/btp324. 19451168 PMC2705234

[10] Garrison E, Marth G. 2012. Haplotype-based variant detection from short-read sequencing. arXiv 1207.3907v2 [q-bio.GN]. doi:10.48550/arXiv.1207.3907.

[11] Shean RC, Makhsous N, Stoddard GD, Lin MJ, Greninger AL. 2019. VAPiD: a lightweight cross-platform viral annotation pipeline and identification tool to facilitate virus genome submissions to NCBI GenBank. BMC Bioinformatics 20:48. doi:10.1186/s12859-019-2606-y. 30674273 PMC6343335

